# Encapsulation and Delivery of the Kinase Inhibitor PIK-75 by Organic Core High-Density Lipoprotein-Like Nanoparticles Targeting Scavenger Receptor Class B Type 1

**DOI:** 10.1021/acsami.4c15472

**Published:** 2024-12-17

**Authors:** Jonathan S. Rink, Adam Y. Lin, Andrea E. Calvert, David Kwon, Alexandra Moxley, Stephen E. Henrich, Aliakbar Mohammadlou, Xu Hannah Zhang, Xiwei Wu, Christiane Querfeld, Donald J. Vander Griend, Hongwei Holly Yin, David A. Horne, SonBinh T. Nguyen, Steven T. Rosen, Leo I. Gordon, Colby Shad Thaxton

**Affiliations:** 1 Department of Medicine, Division of Hematology/Oncology, Northwestern University Feinberg School of Medicine, Chicago, Illinois 60611, United States; 2 Simpson Querrey Institute for Nanotechnology, Northwestern University Feinberg School of Medicine, Chicago, Illinois 60611, United States; 3 Robert H. Lurie Comprehensive Cancer Center, Northwestern University Feinberg School of Medicine, Chicago, Illinois 60611, United States; 4 Department of Urology, Northwestern University Feinberg School of Medicine, Chicago, Illinois 60611, United States; 5 High Throughput Screening Core, City of Hope, Duarte, California 91010, United States; 6 Department of Chemistry, Northwestern University, Evanston, Illinois 60208, United States; 7 Department of Hematology and Hematopoietic Stem Cell Transplantation, Beckman Research Institute, City of Hope, Duarte, California 91010, United States; 8 Department of Computational and Quantitative Medicine, City of Hope, Duarte, California 91010, United States; 9 Department of Pathology, City of Hope, Duarte, California 91010, United States; 10 Department of Pathology, University of Illinois at Chicago, Chicago, Illinois 60612, United States

**Keywords:** lipid nanoparticle, PIK-75, scavenger receptor class B type 1, cancer, targeted delivery, drug encapsulation

## Abstract

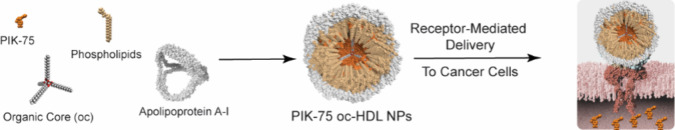

PIK-75 (F7) is a potent multikinase inhibitor that targets p110α, DNA-PK, and p38γ. PIK-75 has shown potential as a therapy in preclinical cancer models, but it has not been used in the clinic, at least in part, due to limited solubility. We therefore developed a nanoparticle to encapsulate PIK-75 and enable targeted cellular delivery. Scavenger receptor class B type 1 (SR-B1) is often overexpressed in cancer compared with normal cells, which enables targeting by synthetic lipid nanoparticles with some features of native high-density lipoprotein (HDL), the natural ligand of SR-B1. We investigated the use of organic core (oc) molecular platforms to synthesize HDL-like nanoparticles (oc-HDL NP). Employing an oc, we successfully formulated PIK-75 into oc-HDL NPs. The PIK-75 loaded oc-HDL NP (PIK-75 oc-HDL NP), comprising ∼20 PIK-75 molecules/NP, has similar size, surface charge, and surface composition as oc-HDL NP and natural human HDL. Using prostate cancer (PCa) and cutaneous T-cell lymphoma (CTCL) models known to be sensitive to inhibitors of p110α and p38γ, respectively, we found that PIK-75 oc-HDL NPs specifically targeted SR-B1 to deliver PIK-75 and potently induced cell death *in vitro* in PCa and CTCL and *in vivo* in a murine PCa model. Additionally, we found that PIK-75 oc-HDL NP, but not free PIK-75 or oc-HDL NP alone, reduced the IC_50_ in the NCI-60 cell line panel and additional pancreatic cancer cell lines. These data demonstrate the first example of drug-loaded oc-HDL NP that actively target SR-B1 and kill cancer cells in vitro and in vivo, encouraging further development and translation to human patients.

## Introduction

The multikinase inhibitor PIK-75, a small molecule inhibitor of the p110α subunit of PI3K, as well as DNA-PK,^1,2^ has been shown to be effective against a wide range of malignancies in the preclinical setting.^3−7^ However, while these results are promising, PIK-75 has not advanced to the clinic due to its poor solubility, bioavailability, biodistribution and pharmacokinetic profile.^8,9^ Our group recently identified the protein kinase p38γ, a member of the MAPK pathway, as a critical mediator for the development and progression in cutaneous T-cell lymphoma CTCL, a disease with limited therapeutic options after first line therapy.^7^ Using a small molecule inhibitor screen, we found that PIK-75 directly bound to p38γ and inhibited its activity.^7^ This inhibition led to CTCL cell death *in vitro* and in an *in vivo* prostate cancer xenograft model. We sought to overcome the obstacles to clinical utility of PIK-75 by developing alternative formulation and delivery strategies using a novel nanoparticle platform.

We have described the synthesis of high-density lipoprotein-like nanoparticles (HDL NP) with size, shape, surface charge and surface chemistry comparable to mature, spherical high-density lipoproteins (HDLs).^10−12^ In some cases, we employ a multiarmed organic core (oc) scaffold molecule as a template to direct the self-assembly of the HDL-defining protein, apolipoprotein A-I (apoA-I), and phospholipids to yield spherical lipoprotein nanoparticles (oc-HDL NPs).^10^ The oc-HDL NPs are ∼15 nm in diameter with a negative surface charge and retain the functional ability to modulate cell cholesterol.^10^ Similar to their natural, spherical HDL counterparts, oc-HDL NPs specifically bind to the high-affinity HDL receptor, scavenger receptor class B type 1 (SR-B1).^10^ We and others have shown that SR-B1 is highly expressed by a wide range of malignancies, including prostate cancer (PCa), breast cancer, lymphoma, glioblastoma, melanoma, ovarian cancer, and others.^13−22^ Targeting SR-B1 with HDL-like particles has been demonstrated as a unique platform to target specific cell types and deliver therapy.^21,23−28^

Overall, we sought to incorporate PIK-75 into oc-HDL NP where the drug can be encapsulated into the more hydrophobic interior of the particles. Successful synthesis of PIK-75 oc-HDL NPs enabled targeted delivery of PIK-75 to cancer cells that highly express SR-B1. The delivery of PIK-75 led to the inhibition of target kinases and induced cancer cell death in vitro and in vivo.

## Results and Discussion

### Synthesis of PIK-75-loaded oc-HDL NPs

The synthesis of oc-HDL NPs was adapted from our original synthesis of oc-HDL NPs where we employed a synthetic tetrahedral phospholipid (PL_4_) as the oc template (PL_4_-HDL NP).^10^ Here, we utilized a novel PE-S_4_ organic core (oc) as a scaffold (Figure S1) and the DOPC phospholipid (Figure S1) to encapsulate PIK-75 (Figure S1). We investigated several stoichiometric combinations of the particle components to assemble oc-HDL NPs of appropriate size and charge and that would accommodate PIK-75. The DOPC phospholipid was selected due to its presence in natural HDLs and cell membranes.^29,30^

**Figure 1:**
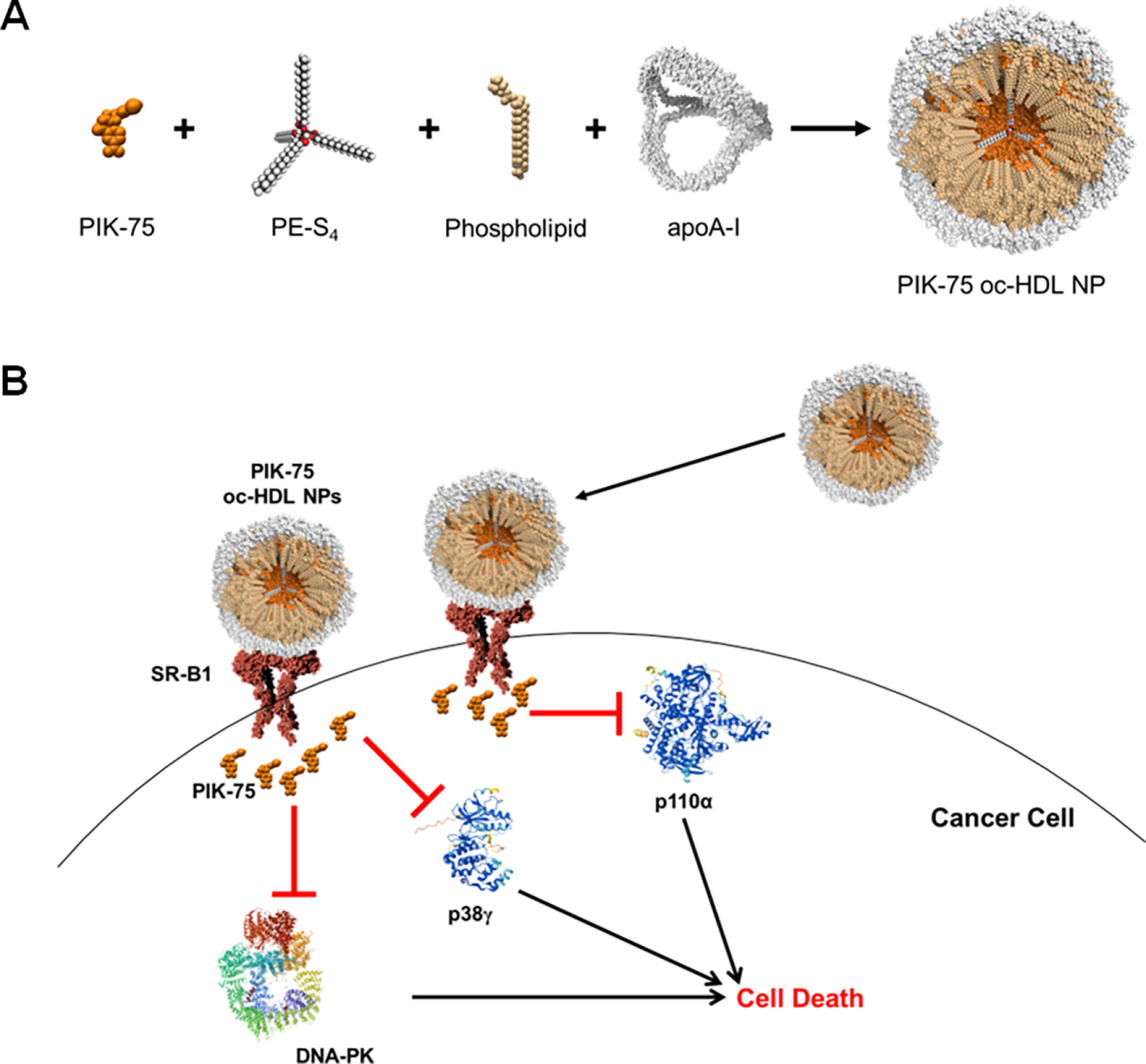
Synthesis scheme and proposed mechanism of PIK-75 oc-HDL NPs. (A) In a single pot synthesis, PIK-75 (DCM), PE-S_4_ organic core scaffold (DCM), phospholipids (DCM), and apoA-I (water) are combined to form an emulsion. The DCM is allowed to evaporate, which results in the spontaneous self-assembly and formation of the PIK-75 oc-HDL NPs. (B) Proposed mechanism-of-action of PIK-75 delivery by oc-HDL NPs. PIK-75 oc-HDL NPs bind to SR-B1 and deliver PIK-75, which can then inhibit various intracellular kinases (p110α, p38γ, DNA-PK) leading to cancer cell death.

The synthesis scheme for PIK-75 oc-HDL NPs is shown in Figure 1. Briefly, the PE-S_4_ oc scaffold, PIK-75, and DOPC phospholipids were dissolved in dichloromethane (DCM). This mixture was added to a solution of apolipoprotein A-I (apoA-I) and sodium cholate in water at a ratio of 3:1 (v/v) water:DCM. A milky white emulsion was generated through successive vortexing and sonication. The DCM was evaporated overnight in a fume hood under constant stirring, which resulted in the formation of the PIK-75 oc-HDL NPs. The same procedure was used to make oc-HDL NPs that were not loaded with PIK-75. In each case, the oc-HDL NPs were purified by centrifugation and dialysis (see Methods). The oc-HDL NPs were characterized for size by DLS and surface charge. Experiments showed that a ratio of 300:25:2:1 phospholipid/PIK-75/apoA-I/organic core yielded PIK-75-oc-HDL NPs of ∼15 nm in diameter with a negative surface charge (Table S1). Additionally, both apoA-I and the core scaffold were required to achieve oc-HDL NPs of desired size (Table S1). PIK-75 oc-HDL NPs had similar size (13.26 ± 4.02 nm in diameter) and negative surface charge (−25.2 ± 0.36 mV) compared to unloaded oc-HDL NPs (11.76 ± 2.03 nm; −22.9 ± 1.60 mV) and natural mature, spherical HDLs.^31,32^ TEM imaging of PIK-75 oc-HDL NPs confirmed the spherical shape and size of PIK-75 oc-HDL NPs (Figure S2).

We next measured the number of apoA-I and PIK-75 molecules per PIK-75 oc-HDL NP. We used BS3^33^ to cross-link intra-NP apoA-I, followed by Western-blot and densitometry analysis, which revealed that each oc-HDL NP had 2–3 molecules of ApoA-I (2.23 ApoA-I/NP; Figure S3). Loading of PIK-75 per nanoparticle was measured by chloroform extraction of PIK-75 followed by UV–vis spectroscopic analysis at 330 nm. Each PIK-75 oc-HDL NP was found to contain 20.3 ± 1.0 PIK-75 molecules, which corresponds to an encapsulation efficiency (EE%) of 72.3% ± 3.7%. The loading capacity (LC%), calculated as the weight of PIK-75 per PIK-75 oc-HDL NP, was 2.91% ± 0.46%. Next, we measured the release of PIK-75 from PIK-75 oc-HDL NPs in an aqueous environment over time. We found that minimal PIK-75 was released from the PIK-75 oc-HDL NPs over 48 h (Figure S4), which indicates that PIK-75 is stably encapsulated and does not leak from oc-HDL NP when in an aqueous environment.

Based on the design and characterization of PIK-75 oc-HDL NPs, we hypothesized that the constructs will bind to SR-B1, deliver PIK-75 and potently induce cell death in cancer cells (Figure 1B).

### PIK-75 oc-HDL NPs Target SR-B1

**Figure 2:**
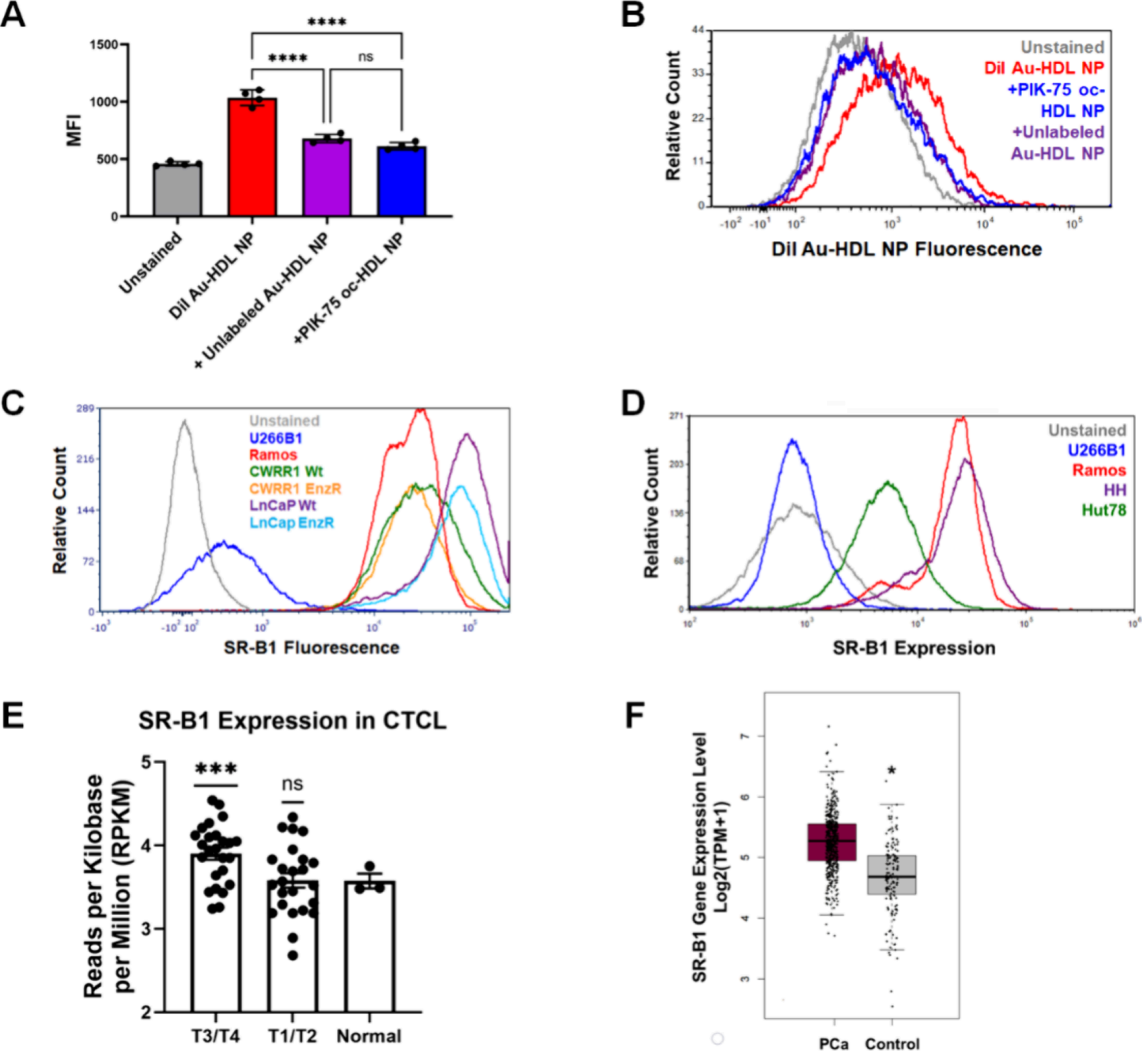
SR-B1 as a target in CTCL and PCa. Competitive binding assay in THP-1 cells. THP-1 cells were treated with fluorescently labeled Au-HDL NPs (DiI Au-HDL NP), and either unlabeled Au-HDL NP or the PIK-75 oc-HDL NP construct for 2 h, followed by flow cytometric analysis. Reduction in fluorescent signal with addition of the unlabeled Au-HDL NP or PIK-75 oc-HDL NPs indicates binding to SR-B1. (A) Median fluorescent intensities (MFI) graph. ****p < 0.0001. *N* = 4 per group. Data displayed as mean ± SD. (B) Representative histogram. (C) Flow cytometry analysis of SR-B1 expression in prostate cancer cell lines cells. Ramos and U266B1 were used as positive and negative controls, respectively. (D) Flow cytometry analysis of SR-B1 expression in HH and Hut78 cell lines. Ramos and U266B1 were used as positive and negative controls, respectively. (E) RNA sequencing analysis of 49 primary CTCL samples demonstrates increased expression of SR-B1 in advanced [stage III (T3) and stage IV (T4)] CTCL compared with normal, CD3+ peripheral T cells. Data (mean ± SD) are presented as reads per kilobase per million (RPKM). ***p < 0.05 by one sample *t* test versus a mean of 1. (F) Comparative analysis of SR-B1 expression in prostate adenocarcinoma sample data were obtained from The Cancer Genome Atlas (TCGA) and Genotype-Tissue Expression database (GTEx). PCa samples (*N* = 492) had significantly higher SR-B1 expression compared to normal prostate tissue (*N* = 152). *p < 0.01 vs PCa.

We employed a competitive binding assay to determine the affinity of the PIK-75 oc-HDL NP to SR-B1. We have previously shown that HDL NPs templated using a 5 nm diameter gold nanoparticle core (Au-HDL NP) specifically target and bind SR-B1 and can be labeled using a fluorescent dye (DiI; DiI Au-HDL NPs).^25,34^ In a competitive binding assay, THP-1 cells, which express SR-B1,^35^ were treated with DiI Au-HDL NPs. Binding of DiI Au-HDL NPs to THP-1 cells resulted in an increase in fluorescence measured using flow cytometry (Figure 2). Co-treatment with unlabeled Au-HDL NPs reduced the fluorescent intensity, as the labeled and unlabeled Au-HDL NPs compete with one another for SR-B1 binding (Figure 2**A, B**). Similarly, cotreatment with PIK-75 oc-HDL NPs and DiI Au-HDL NPs also reduced the fluorescent intensity of THP-1 cells (Figure 2**A, B**), demonstrating that PIK-75 oc-HDL NPs specifically target and bind SR-B1.

### Expression of SR-B1 in CTCL and PCa

We and others have previously reported that SR-B1 is expressed in prostate cancer specimens obtained from patients and in cell lines.^19,20,36−38^ Flow cytometric analysis confirmed SR-B1 expression in the four prostate cancer cell lines [CWRR1 wild type (Wt), CWRR1 enzalutamide-resistant (EnzR), LnCaP Wt, and LnCaP EnzR] used in these studies (Figure 2C).

Expression of SR-B1 has not been previously reported in CTCL. Flow cytometric analysis showed SR-B1 positive staining for the CTCL cell lines HH and HuT78 (Figure 2D). In addition, RNA sequencing data was obtained from 49 primary CTCL samples, grouped by early stage disease (T1/T2; N = 24) and late stage, advanced disease (T3/T4; n = 25), and was compared with normal, CD3+ peripheral T cells (n = 3). SR-B1 expression was increased in T3/T4 CTCL samples compared to control (Figure 2E). Similarly, an analysis of SR-B1 gene expression levels in prostate adenocarcinomas, conducted using The Cancer Genome Atlas (TCGA), and normal prostate tissue, conducted using the Genotype-Tissue Expression (GTEx) database, demonstrated significantly higher expression of SR-B1 in prostate cancer samples (n = 492) compared with normal tissue (N = 152; *p* < 0.01; Figure 2F), confirming previously published reports demonstrating high SR-B1 expression in prostate cancer.^19,20,36−38^ Taken together, these results clearly demonstrate that SR-B1 is a viable target receptor in both CTCL and PCa.

### p110α is the Kinase Target of PIK-75 in Prostate Cancer

Given the ability of PIK-75 to inhibit multiple kinases, including p110α, DNA-PK and p38γ, and its previously demonstrated efficacy against prostate cancer cell lines,^7^ we sought to identify the kinase target of PIK-75 in prostate cancer. Treatment of CWRR1 Wt, CWRR1 EnzR, LnCaP Wt and LnCaP EnzR cells with specific small molecule inhibitors against p110α (A66, Dactolisib, Pictilisib) potently induced cell death, while the DNA-PK inhibitor, NU7441, and the p38γ inhibitor, pirfenidone, were significantly less effective (Figure S5), suggesting that p110α is the kinase target of PIK-75 in prostate cancer.

To confirm these results, the expression of p110α, p38γ and DNA-PK was knocked down using siRNA against each target in the CWRR1 EnzR cell line (Figure S6). Knockdown of p110α significantly decreased cell viability compared with controls (PBS, lipofectamine alone, and scrambled siRNA; Figure S6). Knockdown of DNA-PK marginally reduced cell viability, while p38γ knockdown had only minimal effect. These data confirm that p110α is a critical kinase target in prostate cancer.

### PIK-75 oc-HDL NPs Potently Induce CTCL and PCa Cell Death In Vitro

**Figure 3:**
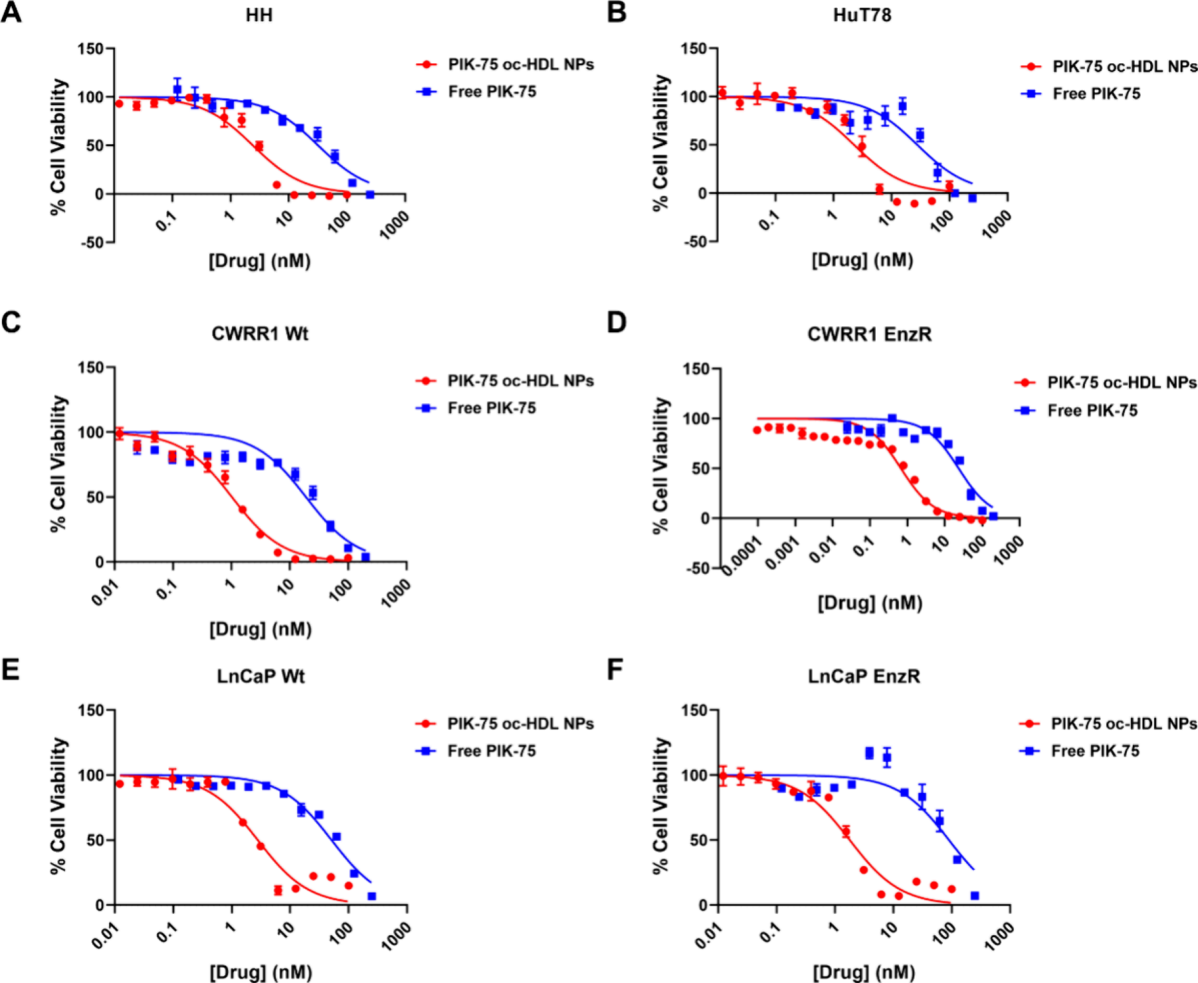
PIK-75 oc-HDL NPs induce cell death in cutaneous T cell lymphoma and prostate cancer cell lines. PIK-75 oc-HDL NPs demonstrated reduced IC_50_ compared with free PIK-75 against the CTCL cell lines HH (A) and HuT78 (B), and the prostate cancer cell lines CWRR1 Wt (C), CWRR1 EnzR (D), LnCaP Wt (E) and LnCaP EnzR (F). *n* = 3 per concentration. Data presented as mean ± SD.

The PIK-75 oc-HDL NPs were tested *in vitro* for efficacy against the CTCL cell lines HH and HuT78 and the prostate cancer cell lines CWRR1 Wt, CWRR1 EnzR, LnCaP Wt and LnCaP EnzR. PIK-75 oc-HDL NPs demonstrated reduced IC_50_ values for all 6 cell lines investigated (IC_50_ for HH = 2.432 nM; IC_50_ for HuT78 = 2.282 nM; IC_50_ for CWRR1 Wt = 1.025 nM; IC_50_ for CWRR1 EnzR = 0.6703 nM; IC_50_ for LnCaP Wt = 2.804 nM; IC_50_ for LnCaP EnzR = 1.802 nM) compared with free PIK-75 (IC_50_ for HH = 32.2 nM; IC_50_ for HuT78 = 28.1 nM; IC_50_ for CWRR1 Wt = 19.0 nM; IC_50_ for CWRR1 EnzR = 23.5 nM; IC_50_ for LnCaP Wt = 51.3 nM; IC_50_ for LnCaP EnzR = 90.0 nM; (Figure 3). Empty oc-HDL NPs had no effect on viability (data not shown).

### SR-B1 Mediates Delivery of PIK-75 by oc-HDL NPs

**Figure 4:**
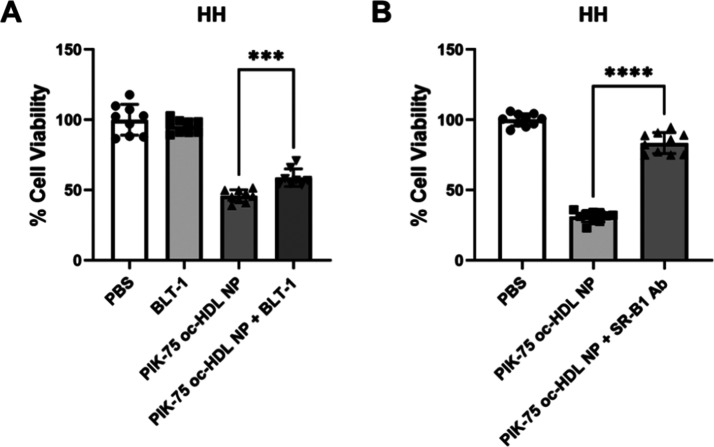
SR-B1 mediates uptake of PIK-75 from oc-HDL NPs. HH cells were pulsed with PIK-75 oc-HDL NPs (50 nM) for 2 h. in the presence or absence of BLT-1 (10 μM), an inhibitor of cholesterol flux through SR-B1 (A), or an SR-B1 blocking antibody (1:100 dilution, B). Addition of BLT-1 ameliorated PIK-75 oc-HDL NP induced cell death compared to PIK-75 oc-HDL NPs alone (A). Similar results were obtained using the SR-B1 blocking antibody (B). (A) *n* = 9 per condition. Data presented as mean ± SD ***p = 0.0001. (B) *n* = 10 per condition. Data presented as mean ± SD ****p < 0.0001.

As noted above, SR-B1 is the target receptor for oc-HDL NPs. To determine if delivery of PIK-75 by oc-HDL NPs occurred through SR-B1, we employed a small molecule inhibitor of SR-B1 activity [blocker of lipid transport-1, BLT-1;^39^] and an SR-B1 blocking antibody.^34^ HH cells were pulsed with PIK-75 oc-HDL NPs with and without BLT-1 (10 μM) or the blocking antibody (1:100 dilution) for 2 h., followed by culture for 72 h. before assessing viability. A short pulse was chosen as PIK-75 oc-HDL NP uptake could potentially occur during nonspecific uptake mechanisms during longer incubations. PIK-75 oc-HDL NPs induced cell death in HH cells, but the addition of BLT-1 (Figure 4A) and the SR-B1 blocking antibody (Figure 4B) rescued cells from PIK-75 oc-HDL NP induced cell death. These results clearly demonstrate that PIK-75 delivery by oc-HDL NPs is mediated by SR-B1.

### Delivery of PIK-75 by oc-HDL NPs Does Not Alter Its Function

**Figure 5:**
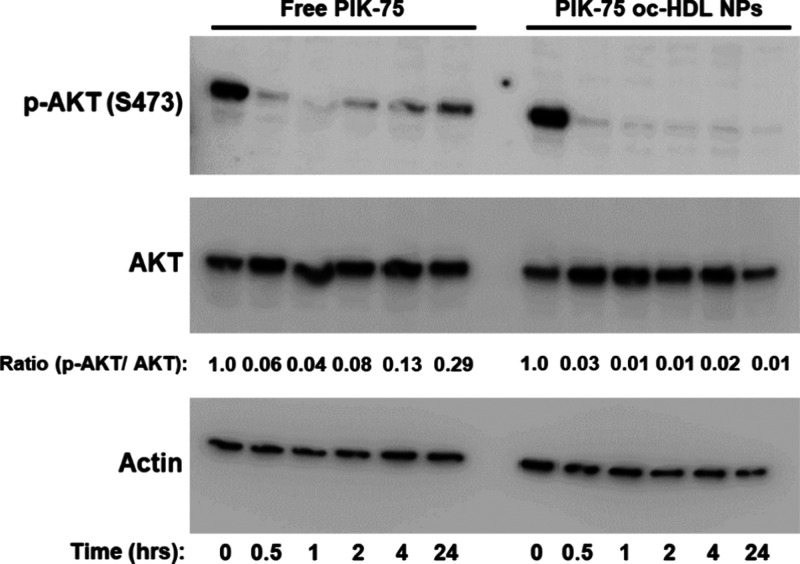
PIK-75 oc-HDL NPs reduce p-AKT levels in prostate cancer cells. Western-blot analysis of CWRR1 EnzR cells treated with free PIK-75 (200 nM; on the left) or PIK-75 oc-HDL NPs (10 nM; on the right) for 0 to 24 h. p-AKT levels rapidly decreased with both free and nanoparticle encapsulated PIK-75. The ratio of p-AKT to AKT was calculated using densitometry with ImageJ.

To determine if delivery of PIK-75 by oc-HDL NPs altered its ability to inhibit its kinase targets, we treated CWRR1 EnzR cells with either free PIK-75 or PIK-75 oc-HDL NPs for up to 24 h, then assayed for the presence of p-AKT, a prominent downstream target of the p110α subunit of PI3K. Treatment with free PIK-75 significantly reduced p-AKT levels from 0.5 to 4 h, with p-AKT signal returning at 24 h (Figure 5). PIK-75 oc-HDL NPs treatment significantly decreased p-AKT signal at 0.5 h, with no recovery observed at 24 h (Figure 5), indicating that delivery of PIK-75 by oc-HDL NPs did not alter the ability of PIK-75 to inhibit p110α, and may in fact enhance its efficacy by prolonging or delaying recovery.

### PIK-75 oc-HDL NPs Have Minimal Effect on SR-B1^+^ Hepatocytes and Macrophages

In order to determine if there was potential toxicity to normal cells expressing SR-B1, we used hepatocytes and macrophages are the two major healthy cell types expressing SR-B1. Treatment of the hepatocyte cell line HepG2 and the macrophage cell line THP-1 with free PIK-75 or PIK-75 oc-HDL NPs did not significantly alter viability in vitro (Figure S7). These data indicate that the major cytotoxic effects of PIK-75 oc-HDL NPs should be confined to the SR-B1^+^ malignant cells.

### PIK-75 oc-HDL NPs Potently Induce Cell Death Across the NCI-60 Panel

To investigate the ability of PIK-75 oc-HDL NPs to induce cell death in malignancies beyond CTCL and PCa, we tested free PIK-75, empty oc-HDL NPs and PIK-75 oc-HDL NPs against the NCI-60 panel of cancer cells, plus 6 additional pancreatic cancer cell lines (FG, BxPC3, Miapaca2, Panc1, CaPan2, AsPC1; Table 1). Free PIK-75 induced cell death, like our previously reported data in CTCL.^7^ Empty oc-HDL NPs did not result in cell death in any cell line tested. PIK-75 oc-HDL NPs potently induced cell death in all the NCI-60 cell lines, with IC_50_ values ranging from 0.42384 nM [HL-60 (TB)] to 16.331 nM (EKVX, Table 1). Three of the pancreatic cancer cell lines (FG, Miapaca2 and BxPC3) were sensitive to PIK-75 and PIK-75 oc-HDL NPs, while the other three pancreatic cancer cell lines (Panc1, CaPan2, AsPC1) were relatively insensitive (Table 1). These data demonstrate that PIK-75 oc-HDL NPs may be effective across a wide spectrum of malignancies.

**Table 1:** Efficacy of PIK-75, oc-HDL NPs, and PIK-75 oc-HDL NPs against the NCI-60 Panel +6 Pancreatic Cancer Cell Lines

**cell line**	**malignancy**	**PIK-75 (nM)**	**PIK-75 oc-HDL NP (nM)**	**oc-HDL NP (nM)**	**PIK-75/PIK-75 NP ratio**
RPMI-8226	Leukemia	29.769	2.0985	>100	14.2
K-562	Leukemia	161.6	4.7915	>100	33.7
MOLT-4	Leukemia	32.049	1.4826	>100	21.6
HL-60 (TB)	Leukemia	26.927	0.42384	>100	63.5
CCRF-CEM	Leukemia	25.054	0.93646	>100	26.8
SR	Leukemia	3.4133	0.80746	>100	4.2
A549/ATCC	NSCLC	22.749	1.6876	>100	13.5
NCI-H460	NSCLC	28.962	1.7131	>100	16.9
EKVX	NSCLC	202.65	16.331	>100	12.4
NCI-H522	NSCLC	27.807	2.5568	>100	10.9
HOP-62	NSCLC	60.58	2.7872	>100	21.7
NCI-H322M	NSCLC	40.001	2.3699	>100	16.9
HOP-92	NSCLC	37.013	1.6657	>100	22.2
NCI-H23	NSCLC	28.292	1.2966	>100	21.8
NCI-H226	NSCLC	113.01	3.5607	>100	31.7
SW-620	Colon	44.62	1.6024	>100	27.8
HT29	Colon	80.729	3.0677	>100	26.3
KM12	Colon	37.709	3.0284	>100	12.5
HCT-116	Colon	15.355	0.85104	>100	18.0
HCT-15	Colon	21.843	1.354	>100	16.1
COLO 205	Colon	24.987	1.2868	>100	19.4
HCC-2998	Colon	36.1	0.8764	>100	41.2
SF-268	CNS	28.215	1.64	>100	17.2
U251	CNS	79.49	2.6394	>100	30.1
SF-295	CNS	75.461	4.1058	>100	18.4
SNB-19	CNS	147.55	5.4134	>100	27.3
SNB-75	CNS	110.82	5.6915	>100	19.5
SF-539	CNS	57.149	2.3387	>100	24.4
MALME-3M	Melanoma	147.29	6.6376	>100	22.2
SK-MEL-2	Melanoma	48.762	3.435	>100	14.2
LOX IMVI	Melanoma	21.248	1.0128	>100	21.0
SK-MEL-28	Melanoma	76.113	3.287	>100	23.2
SK-MEL-5	Melanoma	45.59	3.499	>100	13.0
M14	Melanoma	26.391	2.0471	>100	12.9
MDA-MB-435	Melanoma	48.126	2.7131	>100	17.7
UACC-257	Melanoma	114.36	9.9241	>100	11.5
UACC-62	Melanoma	23.938	1.5317	>100	15.6
NCI/ADR-RES	Ovarian	42.651	2.744	>100	15.5
OVCAR-3	Ovarian	38.769	2.2292	>100	17.4
SK-OV-3	Ovarian	92.944	4.5754	>100	20.3
IGR-OV1	Ovarian	33.237	2.642	>100	12.6
OVCAR-4	Ovarian	167	10.934	>100	15.3
OVCAR-5	Ovarian	39.836	1.9318	>100	20.6
OVCAR-8	Ovarian	27.118	1.7707	>100	15.3
PC-3	Prostate	90.4	2.4237	>100	37.3
DU-145	Prostate	104.83	2.6803	>100	39.1
ACHN	Renal	52.59	3.3433	>100	15.7
UO-31	Renal	22.615	1.0885	>100	20.8
786-O	Renal	49.118	2.3618	>100	20.8
SN12C	Renal	34.677	2.3658	>100	14.7
CAKI-1	Renal	118	2.7255	>100	43.3
TK-10	Renal	120.28	7.777	>100	15.5
RXF 393	Renal	57.022	3.0296	>100	18.8
A498	Renal	72.265	2.3598	>100	30.6
MCF7	Breast	34.98	1.3316	>100	26.3
HS 578T	Breast	26.019	2.3312	>100	11.2
MDA-MB-468	Breast	21.23	1.2032	>100	17.6
MDA-MB-231	Breast	43.809	2.7954	>100	15.7
BT-549	Breast	47.54	0.96736	>100	49.1
T-47D	Breast	268.44	7.0861	>100	37.9
FG	Pancreatic	16.364	1.7437	>100	9.4
BxPC3	Pancreatic	17.42	1.7235	>100	10.1
Miapaca2	Pancreatic	57.451	3.0254	>100	19.0
Panc1	Pancreatic	1214	27.98	>100	43.4
CaPan2	Pancreatic	215.9	18.36	>100	11.8
AsPC1	Pancreatic	>50 μM	>100	>100	N/A

### PIK-75 oc-HDL NPs Reduce Prostate Cancer Tumor Xenograft Growth In Vivo

**Figure 6:**
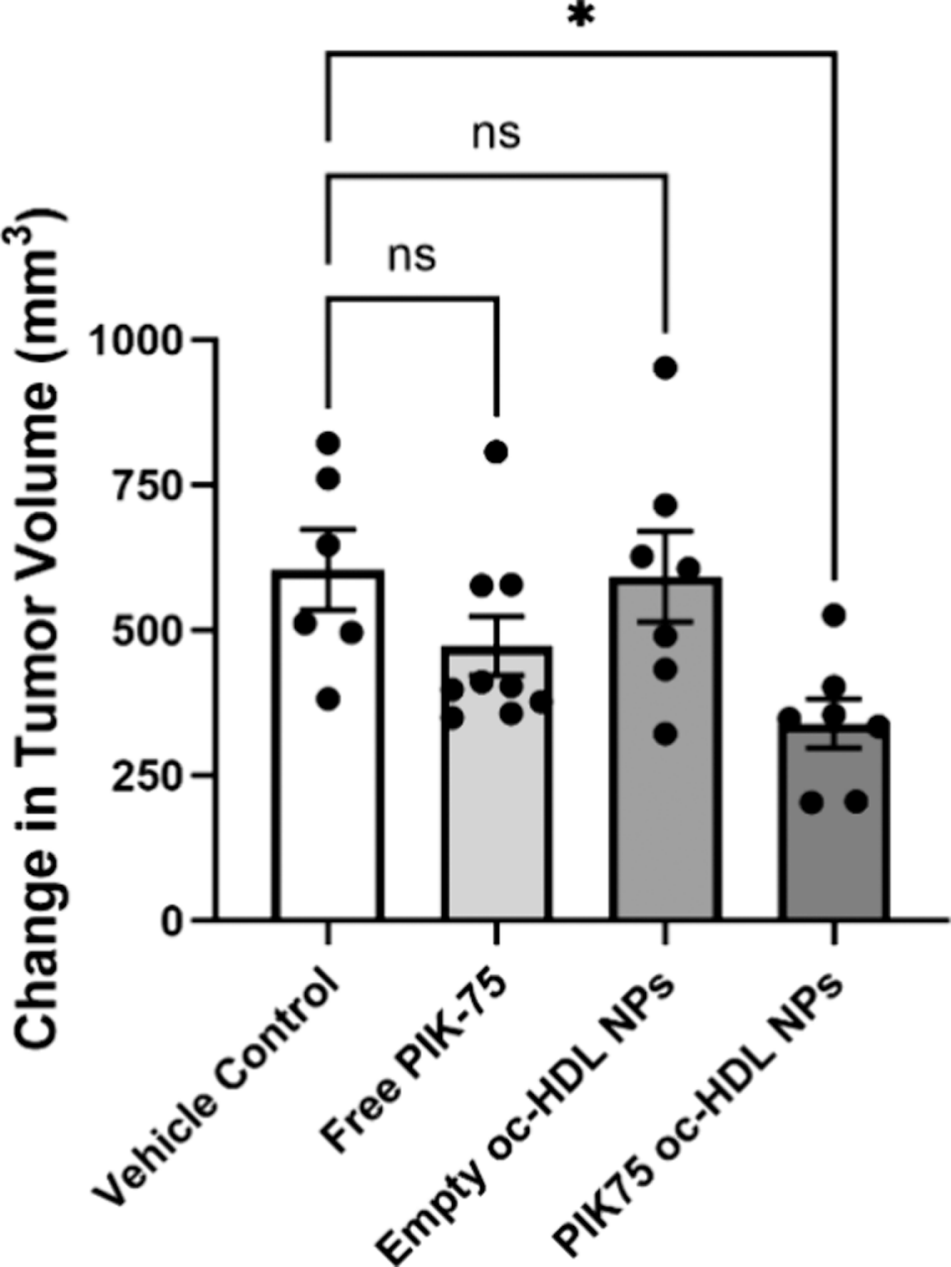
PIK-75 oc-HDL NPs reduce prostate cancer tumor xenograft volumes. CWRR1 EnzR flank tumors were initiated in castrated athymic nude mice, then treated with PBS (200 μL; *n* = 6 mice), free PIK-75 (50 μM, 200 μL; *n* = 9), oc-HDL NPs (2.5 μM, 200 μL; *n* = 7) or PIK-75 oc-HDL NPs (2.5 μM, 200 μL; *n* = 7) i.p. three times per week for 3 weeks. Measurements were taken at the initiation of treatment and end of the study. Data presented as mean change in tumor volume (volume final–volume initial) ± SD. *P* values: vehicle control vs free PIK-75 = 0.3042; vehicle control vs empty oc-HDL NPs = 0.9985; *vehicle control vs PIK-75 oc-HDL NPs = 0.0189.

*In vivo* efficacy of PIK-75 oc-HDL NPs was assayed using a castrate resistant prostate cancer flank tumor model. The CWRR1 EnzR tumor xenografts were initiated in the flanks of immunodeficient male mice that had undergone castration. Intraperitoneal (i.p.) injections of the PIK-75 oc-HDL NP treatments were initiated once tumors were palpable (∼50 mm^3^). We chose the i.p. route of systemic administration as this had been done in a previous study by our group using free PIK-75.^7^ No adverse effects or pain/distress in any of the treatment groups were observed. Treatment with PIK-75 oc-HDL NPs significantly reduced tumor xenograft volume by ∼40% compared with vehicle control (PBS). Free PIK-75 (provided at equimolar concentration of PIK-75 loaded into PIK-75 oc-HDL NPs, based on the 20 PIK-75/NP loading value) and empty oc-HDL NPs did not significantly alter tumor volumes (Figure 6). These data clearly demonstrate the in vivo efficacy of PIK-75 oc-HDL NPs.

## Conclusions

We demonstrate here that the hydrophobic multikinase inhibitor PIK-75 can be successfully encapsulated into oc-HDL NPs. Treatment of cancer cells with PIK-75 oc-HDL NP results in enhanced cell death when compared with free PIK-75 in CTCL, prostate cancer cell lines, a prostate cancer xenograft model, and in the NCI-60 panel plus 6 additional pancreatic cancer cell lines. The PIK-75 oc-HDL NPs targeted SR-B1, which is overexpressed in cancer cells in vitro and in vivo.^13,16,19,20,22,34,40−43^ Delivery of PIK-75 using the oc-HDL NPs did not alter, and may have enhanced, the function of PIK-75. A model is proposed demonstrating stable encapsulation of PIK-75 in oc-HDL NP in aqueous environments whereby the PIK-75 oc-HDL NPs target SR-B1 to deliver PIK-75, inhibit target kinases, and lead to cancer cell death (Figure 1B).

The PIK-75 kinase inhibitor was originally developed as a specific PI3K inhibitor almost 20 years ago,^1^ but it has not been developed clinically due to its poor solubility. Even with this inherent limitation, there is continued interest in PIK-75, due in part to its potent inhibition of multiple kinase targets,^1,2^ and recent data have shown preclinical efficacy against a range of hematologic^4,5^ and solid tumors.^3,6^ Our ability to encapsulate PIK-75 into oc-HDL NP represents the first report using the oc-HDL NP platform to encapsulate and target a cancer therapy to facilitate translation to the clinic.

The PIK-75 oc-HDL NPs specifically target SR-B1, which has been shown to be expressed in both CTCL, PCa, and numerous other malignant cell types *in vitro* and *in vivo.*(13,16,19,20,22,34,40−43) Targeted delivery of PIK-75 potently induced cell death in CTCL and prostate cancer cell lines, as well as in the NCI-60 panel and 6 additional pancreatic cancer cell lines while having minimal effect on normal SR-B1^+^ cells (e.g., macrophages and hepatocytes). Further we found inhibition of tumor growth in a xenograft model in prostate cancer. Accordingly, translation of the PIK-75 oc-HDL NPs to patients may be reasonable for a spectrum of difficult to treat hematologic and solid organ cancers.

PIK-75 delivered by oc-HDL NPs was capable of inhibiting kinase activity, measured by Western blot analysis of p-AKT levels, for a longer duration than free PIK-75. These data indicate that encapsulation of PIK-75 into the oc-HDL NPs may lead to enhanced function and more controlled and prolonged delivery to cancer cells via oc-HDL NP binding SR-B1. Due to the hydrophobicity of the core scaffold molecule and the specific targeting of SR-B1, the oc-HDL NPs are an attractive platform to encapsulate and actively deliver PIK-75 via SR-B1. There are a tremendous number of potential drugs that are hydrophobic in nature, and that may benefit from encapsulation in the oc-HDL NP platform for targeted delivery. Studies have shown that nanoparticle encapsulation and targeted delivery can produce more favorable pharmacokinetics and reduce side-effect profiles for certain therapies in human patients.^44^

The experiments in the expanded NCI-60 cancer cell lines and the 6 pancreatic cell lines provide data that suggest targeted delivery of PIK-75 using the oc-HDL NPs may expand the utility of the PIK-75 oc-HDL NPs to several blood and solid organ cancer types. This will require further preclinical studies, and we are focused on malignancies where there is high unmet clinical need. These data reinforce the findings that PIK-75 is an effective therapeutic against several different malignancies, due to its ability to inhibit critical kinases (e.g., p110α, DNA-PK, p38γ) across a range of signaling pathways. Systemic administration of PIK-75 oc-HDL NPs significantly reduced the growth of PCa xenografts. Taken together, these data provide proof-of-principle that oc-HDL NPs can be leveraged as an active delivery platform for hydrophobic drugs, such as PIK-75, to cancer cells.

## Experimental Section

### Synthesis of PIK-75 oc-HDL NPs

PIK-75 oc-HDL NPs were synthesized using a single-pot synthesis method. The organic core (3-(stearoyloxy)-2,2-bis[(stearoyloxy)methyl]propyl stearate, CAS 115–83–3; PE-S_4_) was purchased from Sigma-Aldrich and used as received. Phospholipids were purchased from Avanti Polar Lipids and apoA-I was purchased from MyBioSource. PIK-75 HCl was obtained from Selleck Chemicals. All other reagents were obtained from Sigma-Aldrich.

For the single pot synthesis, the PE-S_4_ organic core, phospholipid (1,2-dioleoyl-*sn*-glycero-3-phosphocholine, DOPC) and PIK-75 were all resuspended in dichloromethane (DCM), while ApoA-I and sodium cholate were prepared in water. In a glass vial, reagents were combined in a ratio of 300:25:2:1 (DOPC/PIK-75/apoA-I/organic core), and sodium cholate added (final concentration 19 mM), with the final solution composition of 75:25 v/v water:DCM. The solution was then subjected to 3 rounds of alternating vortexing (20 s) and sonication (20 s) to generate an emulsion. The solution was then stirred overnight at room temperature in a chemical fume hood with the vial cap loosened to allow the DCM to evaporate. The solution was then centrifuged for 10 min at 10,000 *g* at 25 °C. The supernatant was collected and dialyzed using a 50 kDa MWCO dialysis tube (G-Biosciences) against a solution of 1 × PBS. The size and surface charge of PIK-75 oc-HDL NPs were measured using dynamic light scattering (DLS) and zeta potential measurements, respectively (Malvern Zetasizer). The concentration of apoA-I was quantified using the BCA protein-quantification assay.

To quantify the number of apoA-I proteins per nanoparticle, we cross-linked the apoA-I on each nanoparticle construct using bis(sulfosuccinidimidyl)suberate (BS3), as described previously.^10,45,46^ Briefly, PIK-75 oc-HDL NPs were diluted to 50 μg protein/mL in PBS. Freshly prepared BS3 was added to each construct (2.5 mM, final). The samples were then allowed to cross-link for 30 min at room temperature. An apoA-I oligomer ladder was prepared by cross-linking purified apoA-I (500 μg protein/mL) using BS3 (0.25 mM) for 4 h at room temperature. For all samples, the cross-linking reaction was halted by addition of 0.5 M Tris base (45 mM, final). Following cross-linking, samples were diluted with 4× Lamelli sample buffer (Bio-Rad), heated for 8 min at 99 °C and ApoA-I oligomers were visualized using Western-blot analysis (see below). To calculate the number of ApoA-I per nanoparticle, densitometry analysis of the various bands (e.g., monomer, dimer, trimer) present in each lane was calculated using ImageJ, as described previously.^10^

The concentration of PIK-75 oc-HDL NPs was determined by BCA protein quantification. The protein content, comprised exclusively of apoA-I, was then converted to molarity (MW apoA-I = 28 kDa) then divided by the number of apoA-I per NP, determined here to be 2.23. To quantify PIK-75 loading into PIK-75 oc-HDL NPs, we first extracted PIK-75 using chloroform. A 1:1 v/v solution of PIK-75 oc-HDL NPs (in 1X PBS) and chloroform was prepared. The solution was vortexed and sonicated (1 min each) to form an emulsion, which was then allowed to settle at room temperature for 1 h. The bottom chloroform layer was then transferred to a quartz cuvette and UV–vis spectrometry was used to measure the absorbance at 335 nm, the wavelength at which PIK-75 displays an absorbance maximum. A standard curve of known PIK-75 concentrations was used to convert the absorbance values to a PIK-75 concentration. Encapsulation efficiency was calculated by dividing the amount of PIK-75 in each nanoparticle by the total amount of PIK-75 added to the synthesis times 100. Loading capacity was calculated by dividing the mass of PIK-75 in the nanoparticles by the nanoparticle weight times 100. Finally, TEM was conducted as described previously.^10^

### PIK-75 Release Assay

To quantify PIK-75 release from oc-HDL NPs in an aqueous solution at 37 °C (physiological conditions), 1 μM PIK-75 oc-HDL NPs were placed into dialysis tubes (50 kDa MWCO, G Biosciences) and dialyzed against 1 X PBS at 37 °C. Samples of the PIK-75 oc-HDL NPs were taken at 0, 1, 4, 8, and 24 h and assessed for PIK-75 content by chloroform extraction and UV–vis spectrometry as described above. Data are reported as percentage of PIK-75 remaining encapsulated in the PIK-75 oc-HDL NPs at each time point.

### Cell Lines

The Ramos (RRID: CVCL_0597), HH (RRID: CVCL_1280), HuT78 (RRID: CVCL_0337), U266B1 (RRID: CVCL_0566), HepG2 (RRID: CVCL_0027) and THP-1 (RRID: CVCL_0006) cell lines were obtained from ATCC and were used within 3 months of receipt and/or resuscitation. The wild-type (Wt) and enzalutamide-resistant (EnzR) clones of CWRR1 (RRID: CVCL_4833 for Wt clone, CVCL_RA55 for EnzR clone) and LnCaP (RRID: CVCL_0395 for Wt clone, CVCL_RA57 for EnzR clone) were generously provided by Dr. Donald Vander Griend at the University of Illinois, Chicago, and were authenticated by ATCC. ATCC uses short tandem repeat (STR) profiling to authenticate the cell lines prior to shipping. The NCI-60 cell line panel and 6 additional pancreatic cell lines (FG, BxPC3, Miapaca2, Panc1, CaPan2 and AsPC1) were obtained from ATCC and used within 30 passages. HuT78 cells were cultured in Iscove’s Modified Dulbecco’s Medium (IMDM, Gibco) supplemented with 20% fetal bovine serum (FBS, Corning) and 1% penicillin/streptomycin (Corning). All other cell lines were cultured in RPMI 1640 (Gibco) supplemented with 10% FBS and 1% penicillin/streptomycin. All cell lines were housed at 37 °C in a humidified, 5% CO_2_ incubator.

### Flow Cytometry Quantification of SR-B1 Expression

Flow cytometry was conducted as described previously.^17,34^ Briefly, lymphoma cell lines (Ramos, HH, HuT78, U266B1) were collected and stained for SR-B1 (antihuman SR-B1, clone m1B9, BioLegend). Cells were then washed and resuspended in FACS buffer (phosphate-buffered saline, 1% bovine serum albumin, 0.1% sodium azide) for flow analysis on the BD Fortessa II flow cytometer (BD Biosciences). Data were analyzed using the FCS Express software.

### SR-B1 Flow Cytometry Binding Assay

Fluorescently labeled (DiI) Au-HDL NPs were synthesized and the SR-B1 competitive binding assay conducted as described previously.^25,34^ Briefly, 5 nm diameter gold nanoparticles (Nanocomposix) were surface functionalized with apoA-I, the phospholipids DPPC (1,2-dipalmitoyl-*sn*-glycero-3-phosphocholine) and PDP PE (1,2-dipalmitoyl-*sn*-glycero-3-phosphoethanolamine-N-[3-(2-pyridyldithio)propionate]; Avanti Polar Lipids), and the intercalating dye DiI (ThermoFisher Scientific). THP-1 cells (2.5 × 10^5^ cells/mL) were incubated with the DiI Au-HDL NPs and an equimolar concentration of PIK-75 oc-HDL NPs or unlabeled Au-HDL NPs for 2 h at 37 °C, 5% CO_2_. Following incubation, cells were centrifuged at 400 *g* for 5 min at 4 °C, washed with ice-cold FACS buffer and resuspended in 400 μL of FACS buffer for flow cytometric analysis on the BD Fortessa II flow cytometer. Data were analyzed using the FCS Express software.

### Western-Blot Analysis

Western blots were conducted as previously described.^17,34^ The Azure 3000 imager was used to image the blots. Antibodies against p110α (ab40776) and DNA-PK (ab133516) were obtained from Abcam, while antibodies against β-actin (#4970), AKT (#9272), p-AKT S473 (#4060) and p38γ (#2307) were obtained from Cell Signaling Technologies. Secondary antibodies were obtained from Bio-Rad. All primary and secondary antibodies were used at a dilution of 1:2000. Densitometry was calculated using ImageJ.

### RNA Sequencing of Primary CTCL Samples

Primary CTCL samples and normal control CD3+ peripheral T cells were collected at City of Hope under an IRB-approved protocol (IRB15328). Data are presented as reads per kilobase per million (RPKM).

### Cell Viability (MTS) Assay

Cell viability was assayed using the MTS assay, as described previously.^17,18,34^ Briefly, CTCL, PCa or normal cells were plated at 16,000 cells/well of a 96 well plate, then treated with PIK-75 oc-HDL NPs, free PIK-75 or vehicle control for 72 h. Following treatment, Promega’s CellTiter Aqueous One solution was added to each well and the absorbance measured at 490 nm using a BioTek Synergy 2 plate reader. Data are presented as relative absorbance (% of control), with vehicle control being set at 100%. Cell viability of the NCI60 panel was conducted and analyzed as described previously.^7^

### NCI-60 Panel

The NCI-60 cell lines (https://dtp.cancer.gov/discovery_development/nci-60/cell_list.htm), selected by the NIH Developmental Therapeutics Program, supplemented with an additional 6 pancreatic cancer cell lines (Miapaca2, Panc1, AsPC1, FG, BxPC3, CaPan2) were used for profiling free PIK-75, empty oc-HDL NPs and PIK-75 oc-HDL NPs. In general, cells were seeded into 384 well tissue culture treated plates overnight and treated with various concentrations (serial of 4-fold dilution from 50 M for free PIK-75, serial of 3-fold dilution from 100 nM for empty oc-HDL NPs and PIK-75 oc-HDL NPs). The CellTiter Glo reagent (Promega) was used to measure viability 72 h after adding drug. IC_50_, defined as the drug concentration that decreases viability by 50%, was calculated using nonlinear regression analysis via GraphPad Prism 10 software (GraphPad Software, Inc.).

### Prostate Cancer Tumor Xenograft Model

All animal work was conducted under an ACUC approved animal protocol in Northwestern University’s Center for Comparative Medicine (CCM). Athymic nude male mice, 10–12 weeks old, were castrated as described previously,^23^ to eliminate the varying levels of androgen production and to mimic the physiologic conditions of patients undergoing androgen deprivation therapy, a common second line treatment for advanced prostate cancer. Following recovery from castration, flank tumors were initiated by seeding 5 × 10^5^ CWRR1 EnzR cells into the right flank. Tumors were allowed to grow to ∼50 mm^3^, at which point the mice were randomized into the following 4 treatment groups: vehicle control; free PIK-75 (200 μL of a 50 μM solution); empty oc-HDL NPs (200 μL of a 2.5 μM solution); and PIK-75 oc-HDL NPs (200 μL of a 2.5 μM solution). Treatments were administered i.p. Three times per week for a total of 9 treatments. Tumor volumes were measured using digital calipers at the end of the treatment cycle.

### Statistical Analyses

Student’s *t* test and one-way ANOVA analyses were used, where appropriate. All statistical analyses were calculated using the GraphPad Prism software.

### Data Availability

The data generated in this study are available on request from the corresponding authors.

## Supporting Information Available

(Figure S1) Structure of the organic core (oc, 3-(stearoyloxy)-2,2-bis[(stearoyloxy)methyl]propyl stearate) scaffold, phospholipid (DOPC, 1,2-dioleoyl-*sn*-glycero-3-phosphocholine), and PIK-75 used to synthesize the PIK-75 oc-HDL NPs; (Figure S2) TEM imaging of PIK-75 oc-HDL NPs; (Figure S3) characterization of the PIK-75 oc-HDL NPs; (Figure S4) PIK-75 is not released from PIK-75 oc-HDL NPs under physiological conditions; (Figure S5) small molecule inhibitors identify p110α as the critical kinase target of PIK-75 in prostate cancer cell lines; (Figure S6) knockdown of p110α in CWRR1 EnzR cells potently induces cell death; (Figure S7) PIK-75 oc-HDL NPs have minimal toxicity on macrophages (THP-1) and hepatocytes (HepG2) cells; (Table S1) composition of PIK-75 oc-HDL NPs (PDF)
