# Supplementary material for: Encapsulation and Delivery of the Kinase Inhibitor PIK-75 by Organic Core High-Density Lipoprotein-Like Nanoparticles Targeting Scavenger Receptor Class B Type 1
Source: ACS Appl Mater Interfaces. 2024 Dec 17;17(1):363–73. doi: 10.1021/acsami.4c15472 (PMC11784712; doi:10.1021/acsami.4c15472)
Supplement: Supplementary file 1 — am4c15472_si_001.pdf [file am4c15472_si_001.pdf]

SUPPORTING INFORMATION

Encapsulation and Delivery of the Kinase Inhibitor PIK-75 by Organic Core High-Density Lipoprotein-Like Nanoparticles Targeting Scavenger Receptor Class B Type 1

Jonathan S. Rink[1,2,3], Adam Y. Lin[1,3], Andrea E. Calvert[2,3,4], David Kwon[5], Alexandra Moxley[4], Stephen E. Henrich[2,3,4], Aliakbar Mohammadlou[6], Xu Hannah Zhang[7], Xiwei Wu[8], Christiane Querfeld[9], Donald J. Vander Griend[10], Hongwei Holly Yin[5], David A. Horne[7], SonBinh T. Nguyen[6], Steven T. Rosen[7], Leo I. Gordon[1,3] and Colby Shad Thaxton[2,3,4*]*

[1]Department of Medicine, Division of Hematology/ Oncology, Northwestern University Feinberg School of Medicine, Chicago, Illinois, 60611, United States.
[2]Simpson Querrey Institute for Nanotechnology, Northwestern University Feinberg School of Medicine, Chicago, Illinois, 60611, United States.
[3]Robert H. Lurie Comprehensive Cancer Center, Northwestern University Feinberg School of Medicine, Chicago, Illinois, 60611, United States.
[4]Department of Urology, Northwestern University Feinberg School of Medicine, Chicago, Illinois, 60611, United States.
[5]High Throughput Screening Core, City of Hope, Duarte, California, 91010, United States.
[6]Department of Chemistry, Northwestern University, Evanston, Illinois, 60208, United States.
[7]Department of Hematology and Hematopoietic Stem Cell Transplantation, Beckman Research Institute, City of Hope, Duarte, California, 91010, United States.
[8]Department of Computational and Quantitative Medicine, City of Hope, Duarte, California, 91010, United States.
[9]Department of Pathology, City of Hope, Duarte, California, 91010, United States.
[10]Department of Pathology, University of Illinois at Chicago, Chicago, Illinois, 60612, United States.

*Leo I. Gordon, MD
Arkes Pavilion, Suite 850
676 N Saint Clair
Chicago, IL 60611
l-gordon@northwestern.edu

*Colby Shad Thaxton, MD, PhD
Lurie Building, 10-118
303 E Superior St
Chicago, IL 60611
cthaxton003@northwestern.edu

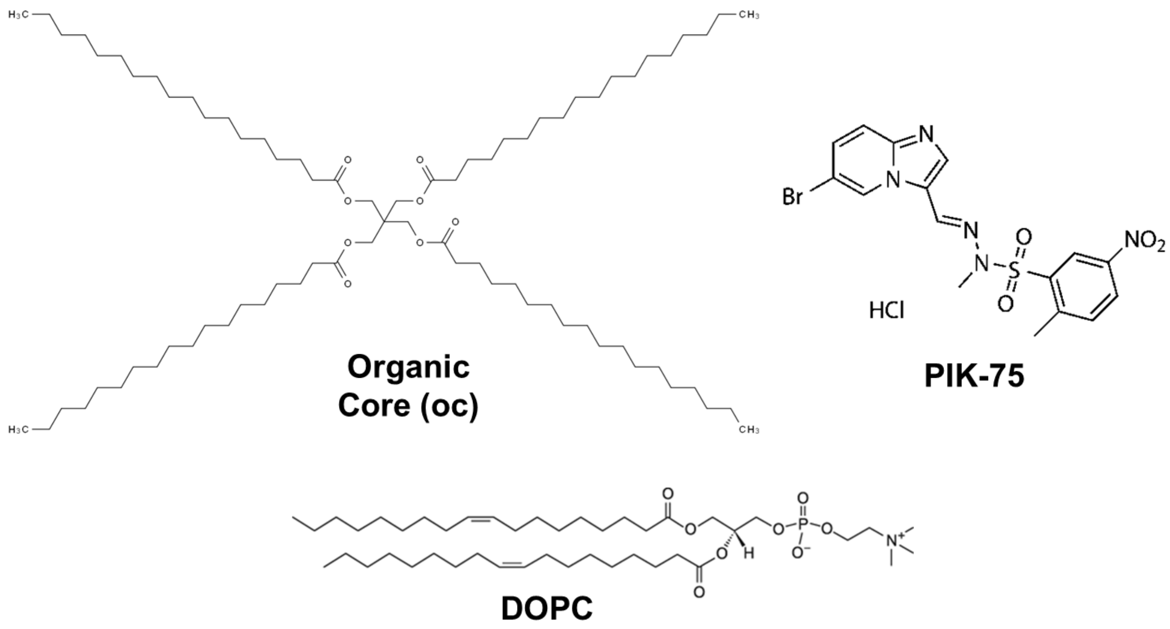

Figure S1. Structure of the organic core (oc, 3-(stearoyloxy)-2,2-bis[(stearoyloxy)methyl]propyl stearate) scaffold, phospholipid (DOPC, 1,2-dioleoyl-sn-glycero-3-phosphocholine), and PIK-75 used to synthesize the PIK-75 oc-HDL NPs.

Table S1. Composition of PIK-75 oc-HDL NPs

Lipid	PIK-75	ApoA-I	Core	Size (d. nm)
300	25	2	1	13.26 ± 4.02
300	25	0	1	25.21 ± 1.51
300	25	2	0	20.41 ± 0.90

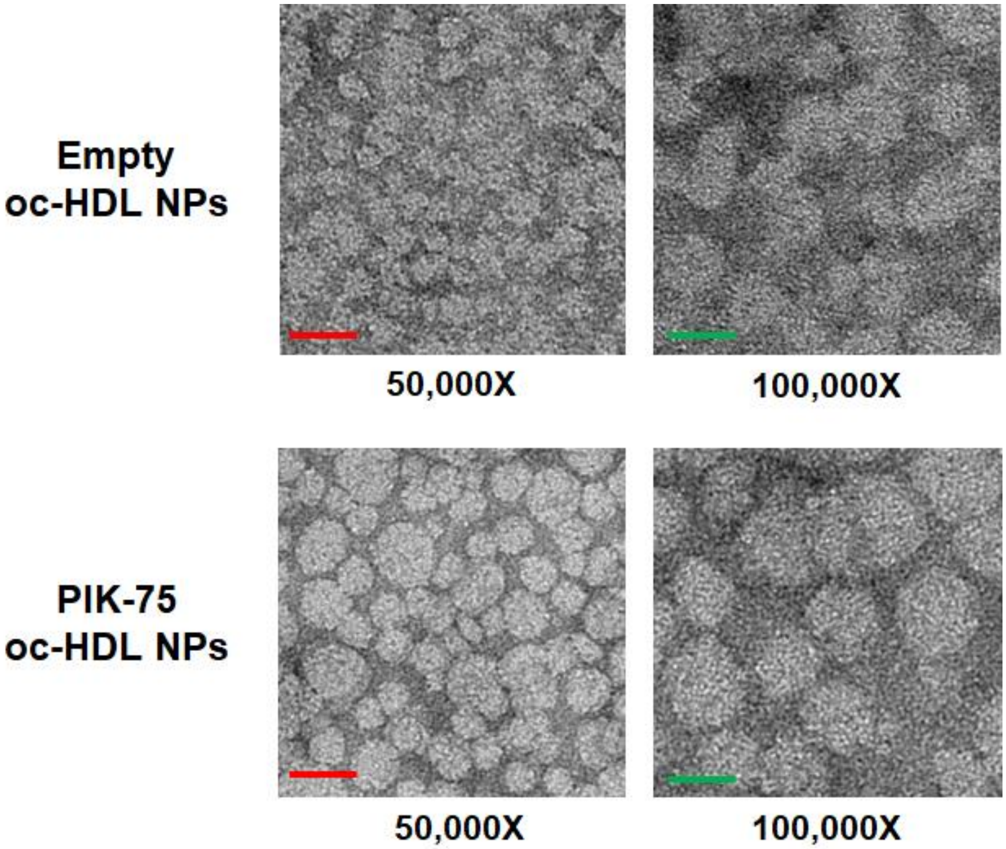

Figure S2. TEM imaging of PIK-75 oc-HDL NPs. Empty oc-HDL NPs (TOP) and PIK-75 oc-HDL NPs (BOTTOM) were imaged at 50,000X (LEFT) or 100,000X (RIGHT). Scale bar (lower left corner) indicates 10 nm for 100,000X magnification and 20 nm for 50,000X magnification.

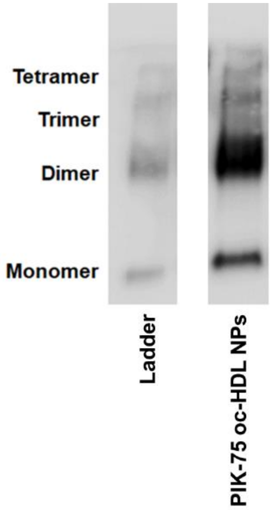

Figure S3. Characterization of the PIK-75 oc-HDL NPs. The ApoA-I on PIK-75 oc-HDL NPs was crosslinked using BS3 and analyzed by Western blot. Densitometry was used to determine the relative prevalence of monomeric, dimeric, trimeric and tetrameric ApoA-I per nanoparticle.

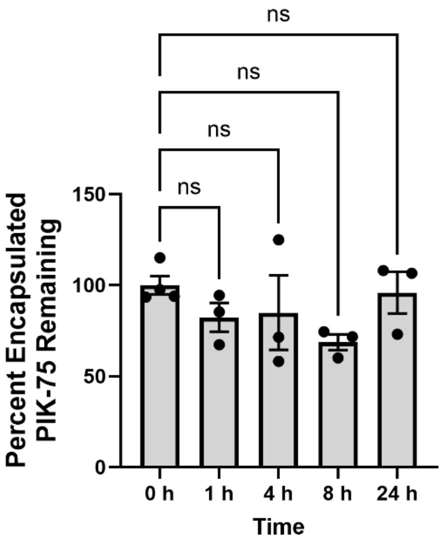

Figure S4. PIK-75 is stably encapsulated in PIK-75 oc-HDL NPs in aqueous conditions. PIK-75 oc-HDL NPs were dialyzed against 1X PBS at 37ºC for 24 hrs. Data are presented as mean ± SEM, with an n = 4 for 0 h and n = 3 for all others.

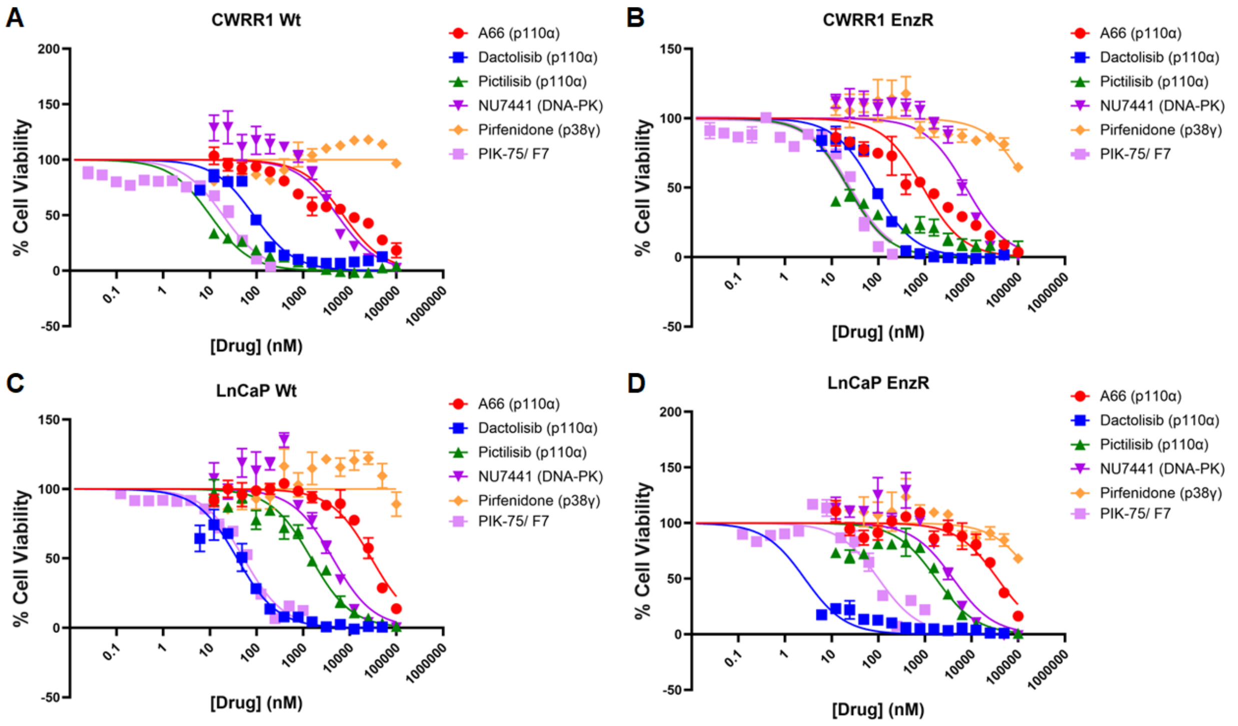

Figure S5. Small-molecule inhibitors identify p110α as the critical kinase target of PIK-75 in prostate cancer cell lines. Prostate cancer cell lines [CWRR1 Wt (A), CWRR1 EnzR (B), LnCaP Wt (C) and LnCaP EnzR (D)] were treated with small-molecule inhibitors of p110α (A66, Dactolisib, Pictilisib), p38γ (Pirfenidone), or DNA-PK (NU7441) for 72 h and assessed for viability by MTS assay. P110α inhibitors potently induced cell death in all four cell lines, while DNA-PK and p38γ inhibitors were significantly less effective.

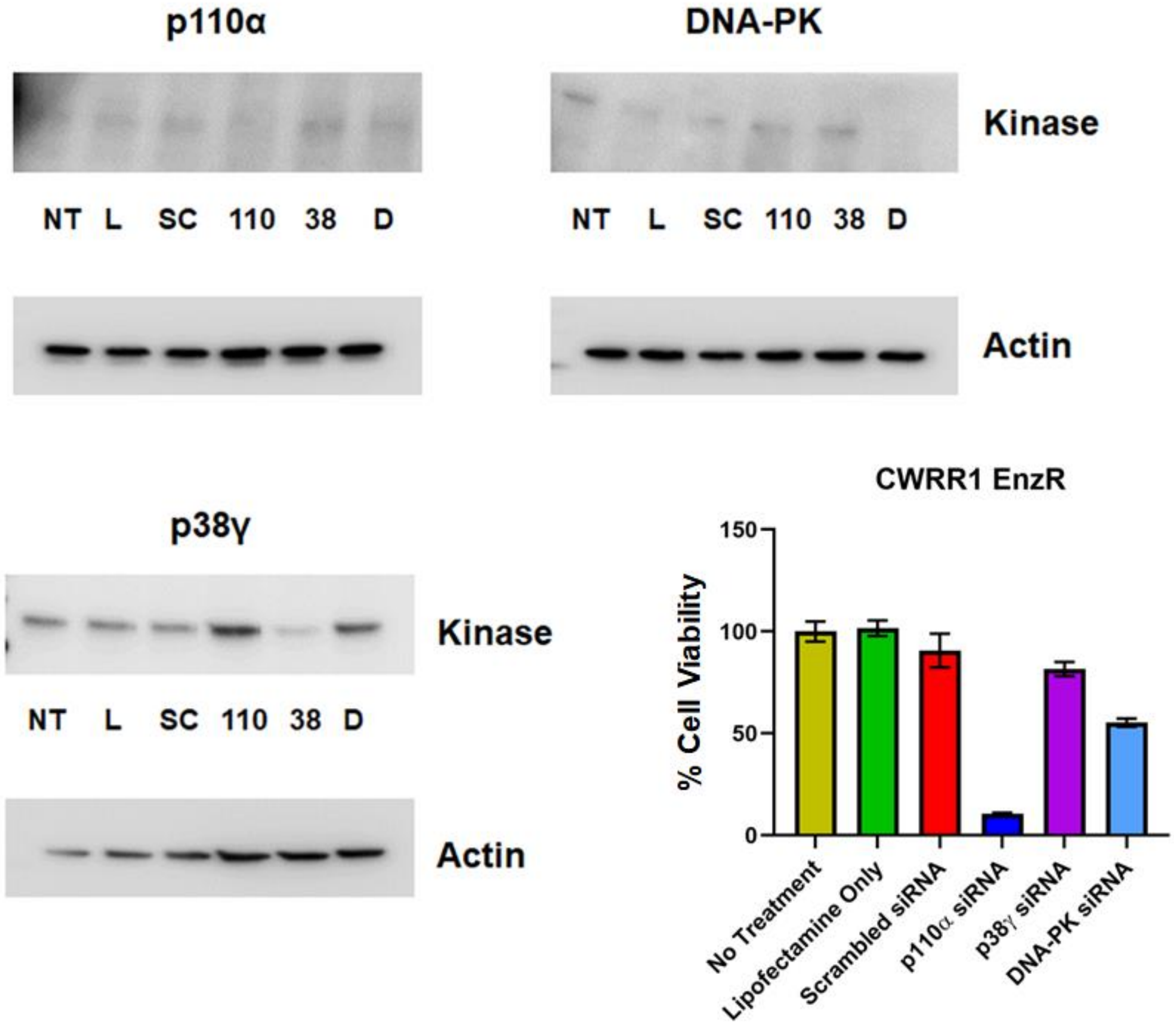

Figure S6. Knockdown of p110α in CWRR1 EnzR cells potently induces cell death. siRNA was used to knock down p110α, p38γ, or DNA-PK in CWRR1 EnzR cells. Western-blot analysis (top, bottom left) confirms knockdown of each target. Knockdown of p110α significantly reduced cell viability (bottom right), while p38γ and DNA-PK knockdown had minimal effect. NT = no treatment; L = lipofectamine only; SC = scrambled siRNA; 110 = p110α siRNA; 38 = p38γ siRNA; D = DNA-PK siRNA. Data are presented as mean ± SD, with an N = 6 per group.

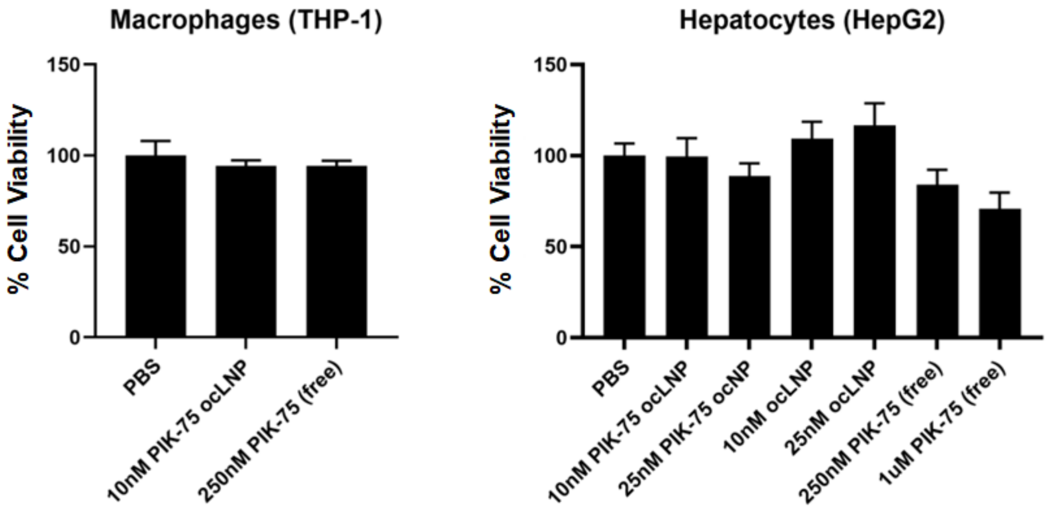

Figure S7. PIK-75 oc-HDL NPs have minimal toxicity on macrophages (THP-1) and hepatocytes (HepG2) cells. Treatment of the macrophage cell line THP-1 and the hepatocyte cell line HepG2 with PIK-75 oc-HDL NPs resulted in minimal cell death, measured using the MTS assay. Data are presented as mean ± SD, with an N = 6 per condition.
